# Justified granulation aided noninvasive liver fibrosis classification system

**DOI:** 10.1186/s12911-015-0181-3

**Published:** 2015-08-06

**Authors:** Marcin Bernas, Tomasz Orczyk, Joanna Musialik, Marek Hartleb, Barbara Błońska-Fajfrowska

**Affiliations:** 1 Institute of Computer Science, Faculty of Computer Science and Material Science, University of Silesia in Katowice, Katowice, Poland; 2 Department of Basic Biomedical Science, School of Pharmacy with Division of Laboratory Medicine in Sosnowiec, Medical University of Silesia in Katowice, Katowice, Poland; 3 Department of Gastroenterology and Hepatology, School of Medicine in Katowice, Medical University of Silesia in Katowice, Katowice, Poland

**Keywords:** Liver fibrosis, Granular computing, Medical support systems, Classification

## Abstract

**Background:**

According to the World Health Organization 130–150 million (according to WHO) of people globally are chronically infected with hepatitis C virus. The virus is responsible for chronic hepatitis that ultimately may cause liver cirrhosis and death. The disease is progressive, however antiviral treatment may slow down or stop its development. Therefore, it is important to estimate the severity of liver fibrosis for diagnostic, therapeutic and prognostic purposes.

Liver biopsy provides a high accuracy diagnosis, however it is painful and invasive procedure. Recently, we witness an outburst of non-invasive tests (biological and physical ones) aiming to define severity of liver fibrosis, but commonly used FibroTest®, according to an independent research, in some cases may have accuracy lower than 50 %. In this paper a data mining and classification technique is proposed to determine the stage of liver fibrosis using easily accessible laboratory data.

**Methods:**

Research was carried out on archival records of routine laboratory blood tests (morphology, coagulation, biochemistry, protein electrophoresis) and histopathology records of liver biopsy as a reference value. As a result, the granular model was proposed, that contains a series of intervals representing influence of separate blood attributes on liver fibrosis stage. The model determines final diagnosis for a patient using aggregation method and voting procedure. The proposed solution is robust to missing or corrupted data.

**Results:**

The results were obtained on data from 290 patients with hepatitis C virus collected over 6 years. The model has been validated using training and test data. The overall accuracy of the solution is equal to 67.9 %. The intermediate liver fibrosis stages are hard to distinguish, due to effectiveness of biopsy itself. Additionally, the method was verified against dataset obtained from 365 patients with liver disease of various etiologies. The model proved to be robust to new data. What is worth mentioning, the error rate in misclassification of the first stage and the last stage is below 6.5 % for all analyzed datasets.

**Conclusions:**

The proposed system supports the physician and defines the stage of liver fibrosis in chronic hepatitis C. The biggest advantage of the solution is a human-centric approach using intervals, which can be verified by a specialist, before giving the final decision. Moreover, it is robust to missing data. The system can be used as a powerful support tool for diagnosis in real treatment.

## Background

Medical diagnosis support systems are gaining acceptance of medical communities and are increasingly used for decision making in difficult diagnostic or therapeutic settings. In this paper, we proposed a medical support system for staging of liver fibrosis based on routine laboratory data.

The research has been carried out on archival data of patients with chronic hepatitis C and reference group of patients with various hepatitis etiologies (hepatitis B virus infection, nonalcoholic steatohepatitis, alcoholic hepatitis), who underwent liver biopsy. The data contains the routine laboratory examinations of peripheral blood, such like blood morphology, coagulation, biochemistry and protein electrophoresis and liver biopsy result. These data, in anonymized form, were obtained from the Dept. of Gastroenterology and Hepatology, Prof. Kornel Gibiński Central Clinical Hospital of the Silesian Medical University in Katowice with the consent of the Head of the Gastroenterology and Hepatology Unit.

Presented approach is used to recognize liver fibrosis stage of patients with hepatitis C virus (HCV) infection. The virus, in the majority of the infected people, is responsible for chronic hepatitis, which ultimately may lead to liver cirrhosis and death. The number of people infected with HCV both in Poland and worldwide is high. According to the Polish National Institute of Hygiene and WHO it is 730 thousand and 150 million [1], respectively. Hepatitis C is a silent disease, hence even 90 % of infected patients are unaware of infection responsible for progressive liver injury. An appropriate pharmacological treatment may slow down or stop a liver damage. Knowledge on the degree of liver fibrosis is important for diagnostic, therapeutic and prognostic reasons. The liver biopsy is still the “gold standard” recommended for assessment of severity of hepatic fibrosis and inflammation, however, this procedure is invasive, expensive, potentially painful and even life-threatening. Moreover, biopsy cannot be repeated in short periods to follow changes in hepatic histology.

Recently, numerous noninvasive biological and physical tests emerged on the medical market conceived to detect advanced hepatic fibrosis and cirrhosis. Unfortunately, the biological tests, like the ELF®[2] or FibroTest®[3], have limited diagnostic specificity [4] and are expensive commercial products. The instrumental methods based on different techniques of measurement of liver stiffness are promising but still not largely introduced to clinical practice [5]. In all validation studies of aforementioned diagnostic methods the liver biopsy is used as the reference technique, however, biopsy itself is an invasive procedure burdened with significant intra- and inter- observer errors [6, 7].

The previous works of authors of this paper [8, 9] have proven that it is possible to estimate liver fibrosis stage, basing on blood tests, but it must be noted that these works were performed on small database. An advantage of the proposed approach over the biopsy is the fact that it does not require hospitalization and can be repeated in regular periods of time without any risk to the patient.

This research is a part of a project aiming to create an on-line E-medical diagnosis support system for non-invasive liver fibrosis recognition [10]. The system should provide an intuitive feedback containing not only a diagnosis, but also its explanation. However, not many solutions offer human-centric approach that would combine reliable classification method with intuitive representation of data. Therefore, granular computing [11, 12] was selected, both from a conceptual as well as algorithmic perspective, in the realization of information model. Using this paradigm, data can be aggregated as many formal representations of information granules: intervals [13], fuzzy sets [14], rough sets [15], shadowed sets [16], or probabilistic sets [17]. There are several works, which prove the usefulness of granular concept [18–20]. The effectiveness of the proposed method was compared with other, most commonly used solutions – the *k*-nearest neighborhood (kNN) variants [21], perceptron neural network [22], Radial Basis Function network [23], random trees [24] and naive Bayes classifier method [25].

## Methods

The method was built based on a data set containing 33 different blood attributes collected from 290 chronic viral hepatitis patients from the Dept. of Gastroenterology and Hepatology of the Prof. Kornel Gibiński Central Clinical Hospital of the Silesian Medical University in Katowice as well as 75 patients with other hepatitis etiologies. Due to high count of missing values (over 66 %) in some attributes, eight of them have been eliminated from the set, leaving 25 of them (see Table 1) for further processing. Eliminated attributes did not have values representation for all stages of liver fibrosis.

**Table 1:** Data set characteristics

No (k)	Parameter [unit]	Mean (std. deviation)	Missing values	No (k)	Parameter [unit]	Mean (std. deviation)	Missing values
1	Age [years]	57,4 (14,15)	0 %	14	GGT [IU/L]	70.9 (66.15)	3 %
2	Hemoglobin [g/L]	14.6 (1.71)	58 %	15	Creatinine [mg/dL]	1.0 (0.35)	60 %
3	RBC [10^6^/UL]	4.8 (0.62)	58 %	16	Glucose [mg/dL]	96.4 (19.83)	62 %
4	WBC [10^3^/UL]	6.1 (1.9)	0 %	17	Na [mmol/L]	138.3 (3.10)	63 %
5	PLT [10^3^/UL]	197.1 (59.5)	0 %	18	K [mmol/L]	4.3 (0.46)	63 %
6	PT [sec.]	12.0 (4.7)	27 %	19	Cholesterol [mg/dL]	187.0 (38.71)	20 %
7	PTP [(%]	99.6 (15.75)	3 %	20	Total protein [g/dL]	7.5 (0.64)	16 %
8	APTT [sec.]	33.5 (5.59)	42 %	21	Albumin [g/dL]	0.5 (0.25)	29 %
9	INR	1 (0.11)	12 %	22	Albumin [%]	60.9 (5.92)	23 %
10	AST [IU/L]	63.8 (48.54)	1 %	23	Globulin α_1_ [%]	2.7 (0.87)	24 %
11	ALT [IU/L]	82.5 (64.26)	0 %	24	Globulin α_2_ [%]	9.2 (1.53)	24 %
12	ALP [IU/L]	80.3 (29.99)	5 %	25	Globulin β [%]	10.6 (1.70)	24 %
13	Bilirubin [mg/dL]	1.0 (0.64)	6 %	26	Globulin γ [%]	16.4 (5.09)	23 %

RBC- Red blood cells; WBC- White blood cells; PTL- Platelets; PT- prothrombin time; PTP- prothrombin ratio; APTT- activated partial thromboplastin time; INR- international normalised ratio; AST- aspartate aminotransferase; ALT- alanine aminotransferase; ALP- alkaline phosphatse; GGT- γ-glutamyltransferase; Na- natrium; K- kalium

A set of patients’ blood attributes (*K*) has been determined as shown in Table 1. Based on previous research [10], patients’ age has been included as an additional attribute, thus the set *K* contains 26 attributes. Variable *k* = 1, …, 26 is used to denote the number of an attribute.

For every patient, the biopsy results have been collected as a reference value. Liver biopsy examination was performed according to the METAVIR scoring system [31]. Fibrosis level was staged on a range of 0–4 with step 1: F0 – no fibrosis, F1 – portal fibrosis without septa, F2 – few fibrosis, F3 – numerous septa without cirrhosis and F4 – cirrhosis.

Many authors point out that some biopsy fibrosis stages are difficult to diagnose, even for experienced doctors [10]. For this reason, after medical consultations, the new classification has been introduced. Instead of F0 and F1 fibrosis stage, the low (n = 1) level class S_1_ was applied. Instead of F2 and F3, the medium (n = 2) level class S_2_ was used, while instead of F4 METAVIR cirrhosis, the S_3_ class (n = 3) was applied. It means that instead of five METAVIR scoring scale, the three *S*_*n*_*, n* = 1, …, 3 fibrosis classes will be taken into consideration. The new classification scores were introduced to the proposed medical support system.

**Fig. 1:**
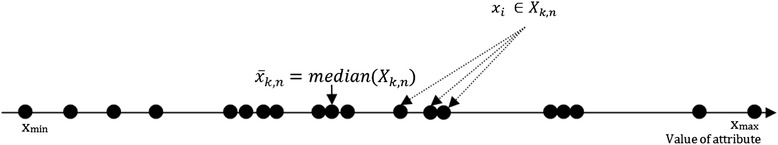
The illustration of *X*_*k,n*_ set for a given *k* and *n* in a value domain

Blood and age data were grouped according to the biopsy result and assigned to the sets *X*_*k*,*n*_, where *k* = 1, …, 26 is a given blood attribute and the number *n* = 1, …, 3 describes one of the new fibrosis *S*_*n*_ classes. The data is processed for every *k*^*th*^ blood and age attribute separately, so every set *X*_*k*,*n*_ represents the *n*^*th*^ fibrosis class of *k*^*th*^ attribute. Each set *X*_*k*,*n*_ includes up to *P* values, where *P* is a number of examined patients. For example the set *X*_10,3_ comprises values *x*_*i*_, *i* = 1, …, *P*^'^*P*^'^ ≤ *P* of the blood attribute *k* = 10 (ASPT) of all patients, who were diagnosed with fibrosis class n = 3 (*S*_*3*_). It means that theoretically we can create *k* × *n* = 26 × 3 = 78 various *X*_*k*,*n*_ sets. An exemplary set *X*_*k*,*n*_ is presented in Fig. 1.

Due to missing values in patients’ data, the cardinality of the *X*_*k,n*_ sets is various. Therefore, the granulation process is focused on the values distribution within a set and not cardinality itself.

**Fig. 2:**
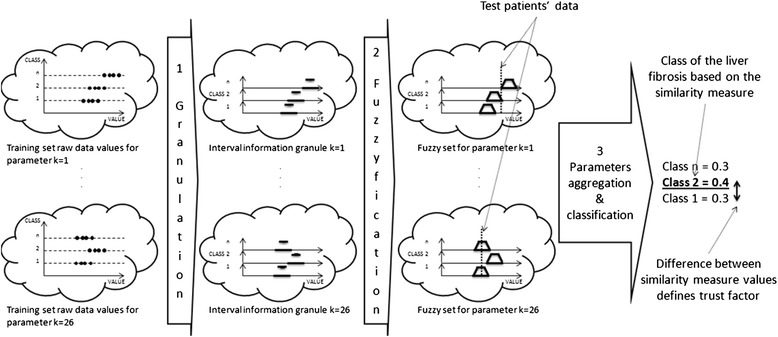
Data representation during classification process: clouds of black points (elements of *X*_*k,n*_ sets), series of intervals and fuzzy sets. The intuitive classification algorithm merges the obtained results using voting procedure

In the proposed method, medical data are processed to acquire useful information. The medical data processing is realized inside of three functional blocks. The first block, using *X*_*k*,*n*_ sets, creates intervals based on the justified granulation paradigm. This transition is shown in the left part of Fig. 2. The clouds of black points are described as a series of intervals. In the middle block the intervals are generalized to fuzzy sets using fuzzification procedure. Finally, the intuitive classification algorithm is proposed to merge the obtained results using voting procedure, which is a common approach in advanced biometric systems [26]. The system’s functional diagram in Fig. 2 presents information flow and changes of medical data representation.

### Information mining using justified granulation method

As mentioned before, due to missing data, the cardinality of *X*_*k,n*_ sets varies. Therefore, the granulation process is focused on the values distribution within a set and not cardinality itself. Direct analysis of the raw blood and age attributes for fibrosis stage evaluation could be troublesome, therefore the justified granularity paradigm [27] was adopted for this task. This data mining technique creates an interval granule over a set *X*_*k,n*_*.* To find an interval representation over a *X*_*k,n*_ set, its left and right boundary is determined using information function *V* family, defined in Eq. 1:

1 $$ \begin{array}{l}{V}_r\left({x}_i,{X}_{k,n},\alpha \right)={f}_1\left( card\left(\left\{{x}_j\in {X}_{k,n}:{\overline{x}}_{k,n}<{x}_j\le {x}_i\right\}\right)\right)\times {f}_2\left(\left|{\overline{x}}_{k,n}-{x}_i\right|\right),\\ {}{V}_l\left({x}_i,{X}_{k,n},\alpha \right)={f}_1\left( card\left(\left\{{x}_j\in {X}_{k,n}:{\overline{x}}_{k,n}<{x}_j\ge {x}_i\right\}\right)\right)\times {f}_2\left(\left|{\overline{x}}_{k,n}-{x}_i\right|\right),\end{array} $$

where:

$$ {\overline{x}}_{k,n}= median\left({X}_{k,n}\right), $$

$$ {f}_1(u)=u, $$

$$ {f}_2(z)= \exp \left(-\alpha z\right), $$

*X*_*k,n*_ - set contains values of *k*^*th*^ blood attribute of patients with the *n*^*th*^ fibrosis class,

${\overline{x}}_{k,n}$ - median over a set *X*_*k,n*_,

*α*- specificity parameter *α* = [0, *α*_*max*_].

**Fig. 3:**
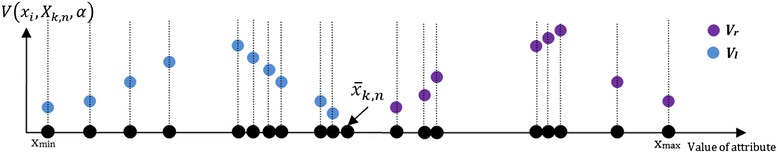
The illustration of *X*_*k,n*_ set for given *α* in value domain. The functions *V*_*l*_ and *V*_*r*_ assume maximal values in proximity of local concentration of groups of elements of the set

The intuitive character of *V* family function was illustrated in Fig. 3.

If the elements of the set are uniformly distributed then the maximum of *V* function is directly affected by *α* value. The functions *V*_*l*_ and *V*_*r*_ favor the boundary values of a set for *α* = 0 to values close to median for *α = α*_*max*_. In practice, for values *α* ∈ (0, *α*_*max*_), the *V* function family assumes maximal values in proximity of local concentration of groups of elements of the set. These values can be treated as characteristic representation of a set for a given *α*. The balance between cardinality of the set and concentration of values inside of this set can be tuned using *α* parameter. Using defined *α* value the representation of information granule *g*_*k,n,α*_ = *G*(*X*_*k,n,*_*α*), as interval [*a*_1,*k*,*n*,*α*_, *a*_2,*k*,*n*,*α*_] can be determined by finding the values for both *v*_*l*_ and *v*_*r*_ functions according to Eq. 2.

2 $$ \begin{array}{l}{g}_{k,n,\alpha }=G\left({X}_{k,n,\alpha}\right)=\left[{a}_{1,k,n,\alpha },{a}_{2,k,n,\alpha}\right]\\ {}{a}_{1,k,n,\alpha }={v}_l\left({X}_{k,n},\alpha \right){=}_{x_{i\in }{X}_{k,n}}^{\arg max\kern0.5em \left\{{V}_l\left({X}_{k,n},{x}_i,\alpha \right)\right\}}\\ {}{a}_{2,k,n,\alpha }={v}_r\left({X}_{k,n},\alpha \right){=}_{x_i\in {X}_{k,n}}^{\arg max\kern0.5em \left\{{V}_r\left({X}_{k,n},{x}_i,\alpha \right)\right\}}\end{array} $$

Before going to the next stage, the specificity parameter *α* value should be normalized from [0, *α*_*max*_] to [0,1]. The normalization procedure was described thoroughly in [27, 32].

**Fig. 4:**
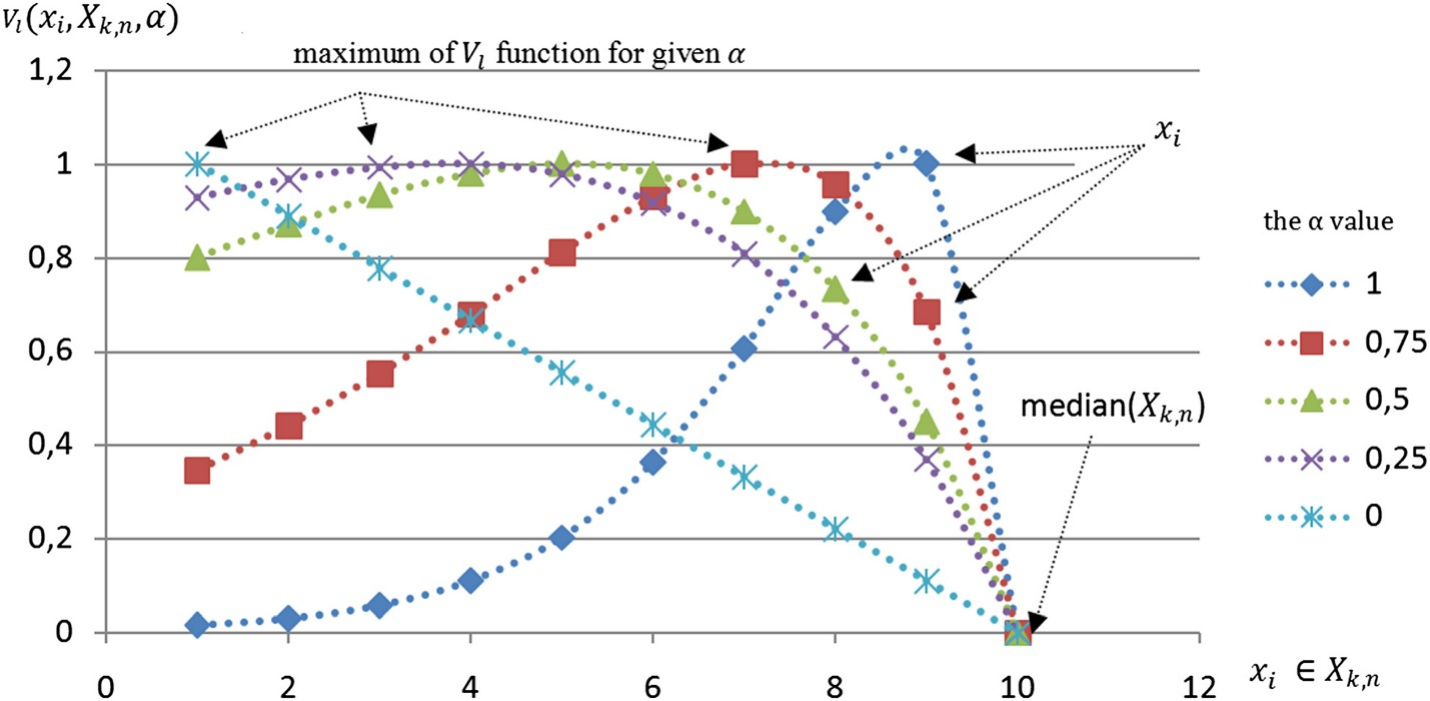
The example of normalized *V*_*l*_ function for various α value. The maximal value of *V*_*l*_ function, for given α, allows to find a local concentration of elements within *X*_*k,n*_ set

Eq. 2 provides a balance between the specificity of a granule and its size. The advantage of proposed method over other solutions is only one parameter to tune. Moreover, the *α* value influences the area where the group of values is searched. The interval representing a set is found by its left and right boundary according to Eq. 2. An example of *V*_*l*_ functions family, which values was normalized to [0,1], is shown in Fig. 4.

The *v*_*l*_ function values represent maximal values of *V*_*l*_ function for given *α*, thus allows to find a local concentration of elements within *X*_*k,n*_ set. The granulation algorithm (*G*), which finds an interval representation for a given *α* and *X*_*k,n*_ set, is defined as follows:

1. Calculate value *a*_*1*_*,*_*k,n,α*_ = *v*_*l*_ (*X*_*k,n*_, α).
2. Calculate value *a*_*2,k,n,α*_ = *v*_*r*_ (*X*_*k,n*_, α).
3. Construct information granule *g*_*k,n,α*_ *=* [*a*_*1,k,n,α*_, *a*_*2,k,,n,α*_].

The granulation algorithm processes elements of a set to find its representation. Therefore, performing *z* ∈ *Nα* -cuts equally distributed within a range [0,1], will allow to finding a characteristic values for each set. To find the pattern within all ranges of specificity level, as illustrated in Fig. 4, the series of *z* interval granules for *α*_*i*_, *i* ∈ {0, …, *z* − 1} are built, where values of α_*i*_ are equally distributed within [0,1]:

3 $$ {\alpha}_i=\left\{\begin{array}{lll}0\hfill & :\hfill & z=1\hfill \\ {}\frac{i}{z-1}\hfill & :\hfill & z>1\hfill \end{array},i\in \left\{0,\dots, z-1\right\}\right\} $$

**Fig. 5:**
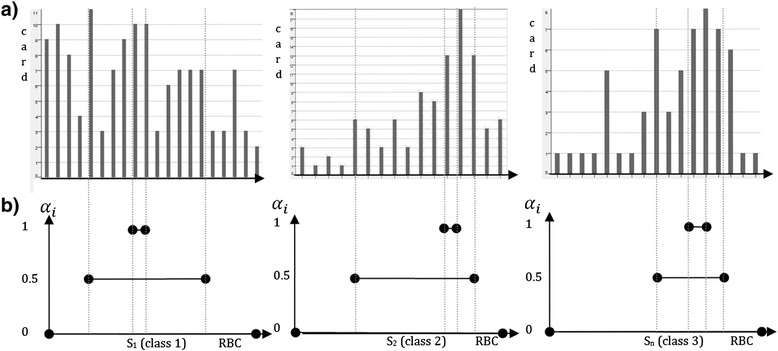
The RBC attribute interval representation for class 0, 1 and 2 respectively: (**a**) histogram (**b**) generated interval granules for *z* = 3

The result for parameter *z* = 3 is a sequence of three granules generated for *α*_*i*_ ∈ {0, 0.5, 1}.The example for blood RBC attribute, in comparison with histogram, is presented in Fig. 5 for each class.

The proposed approach does not favor the class with higher number of samples. Moreover, the received intervals illustrate a change within a set. Intervals are crisp and do not take under consideration values which are lying in close proximity to its boundaries. Thus, fuzzification was proposed in next step to consider this feature.

#### Interval fuzzification procedure

The process of changing interval representation of information granule to fuzzy set adds an uncertainty level. Based on two interval information granules constructed over set *X*_*k,n*_ (${g}_{\mathsf{k},\mathrm{n},{\mathit{\mathsf{\alpha}}}_{\mathit{\mathsf{0}}}}\mathrm{and}\ {g}_{\mathsf{k},\mathrm{n},{\mathit{\mathsf{\alpha}}}_{\mathit{\mathsf{j}}}}$, where j ∈ {0, …, *z* − 1}), a fuzzy set granule ${\overline{g}}_{k,n,{a}_0},{\alpha}_j$ is build. In the proposed method, the *α*_0_ is constant value equals 0 and it represents all values of the *X*_*k,n*_ set. The granulation function $\tilde{G}$, which constructs fuzzy granule and its membership function, was defined as follows:

4 $$ \tilde{G}:\left({g}_{\mathrm{k},\mathrm{n},{\alpha}_0},{g}_{\mathrm{k},\mathrm{n},{\alpha}_{\mathrm{j}}}\right)\to {\tilde{g}}_{k,n,{\alpha}_0,{\alpha}_j}, $$

$$ {\tilde{g}}_{k,n,{\alpha}_0,{\alpha}_j}\equiv {\tilde{g}}_{k,n,0,{\alpha}_j}=\left({b}_1,{b}_2,{b}_3,{b}_4\right), $$, where:

$$ {b}_1= \inf \left({\mathit{\mathsf{g}}}_{k,n,0}\right)={a}_{1,k,n,0}, $$

$$ {b}_2= \inf \left({\mathit{\mathsf{g}}}_{k,n,{\alpha}_{\mathrm{j}}}\right)={a}_{1,k,n,{\alpha}_j}, $$

$$ {b}_3= \sup \left({\mathit{\mathsf{g}}}_{k,n,{\alpha}_j}\right)={a}_{2,k,n,{\alpha}_j}, $$

$$ {b}_4= \sup \left({\mathit{\mathsf{g}}}_{k,n,0}\right)={a}_{2,k,n,0} $$

$$ {\mu}_{{\tilde{g}}_{k,n,0,{\alpha}_j}}\left(x,d\right)=\left\{\begin{array}{lll}\frac{x-{b}_1\left(1-d\right)}{b_2-{b}_1\left(1-d\right)}\hfill & :\hfill & x\ge {b}_1\left(1-d\right)\kern1em  and\kern0.5em x<{b}_2\hfill \\ {}\frac{b_4\left(1+d\right)-x}{b_4\left(1+d\right)-{b}_3}\hfill & :\hfill & x\le \left(1+d\right)\kern1em  and\kern0.5em x\kern0.5em >\kern0.5em {b}_3x\in \boldsymbol{R},d\in \left[0,1\right]\hfill \\ {}1\hfill & :\hfill & x\ge {b}_2\kern0.5em  and\kern0.5em x\le {b}_3\hfill \\ {}0\hfill & :\hfill & otherwise\hfill \end{array}\right., $$

where:

*k-* number of an attribute,

*n*- liver fibrosis class,

*d* - generalization parameter *d* ∈ [0, 1],

*α*_*j*_- *j*^th^ α-cut evaluated using Eq. 3, *j* ∈ {0, …, *z* − 1},

*α*_0_- constatnt equal 0 in the equation,

*z* - number of cuts,

inf/ sup - lower/upper boundary of an interval (*g* granule).

**Fig. 6:**
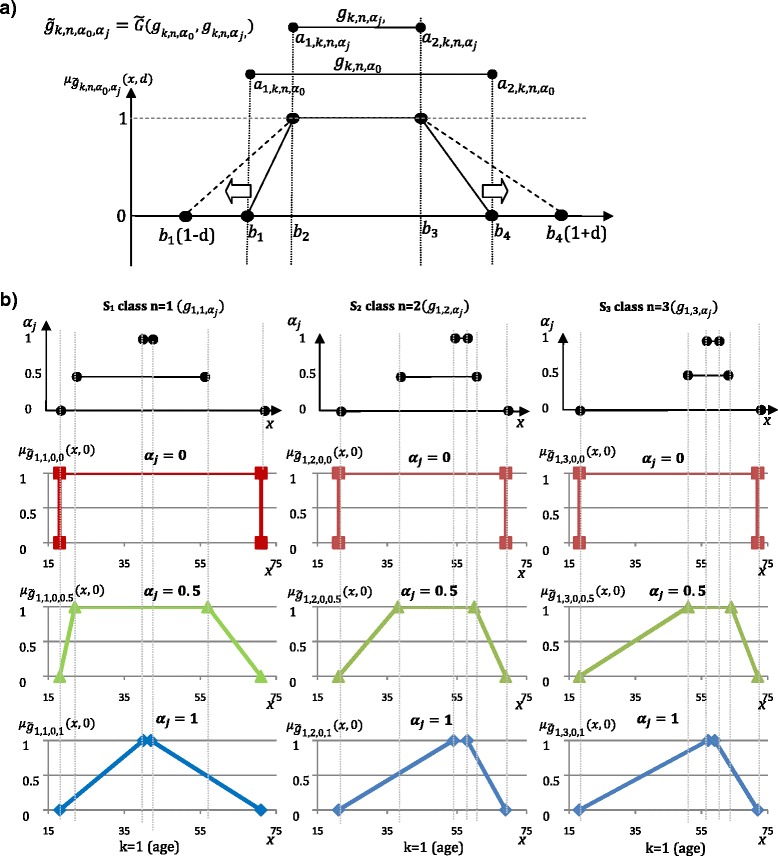
$\tilde{g}$ membership function for a given *αj* and changing *d* parameter: (**a**) example, (**b**) real estimated data for Age attribute

The proposed granule ${\tilde{g}}_{k,n,0,{\alpha}_j}$ describes the *n*^*th*^ class of liver fibrosis for a given *k*^*th*^ attribute and *j*^th^ α-cut. The boundary values of ${\tilde{g}}_{k,n,0,{\alpha}_j}$ are represented by constant *α*_*0*_ = 0, therefore *b*_*1*_ and *b*_*4*_ will always assume respectively minimal and maximal value of *X*_*k,n*_ set. The trapezoidal fuzzy membership function *μ*(*x*) was selected as intuitive fuzzy representation of two intervals. Moreover, this set representation simplifies the calculations and was successfully applied in many medical works [28, 29]. Finally, the initial experiments with other fuzzy representation e.g. Gaussian, triangle, bell-shape did not have impact on model accuracy. The introduced in Eq. 4 generalization parameter (*d*), illustrated in Fig. 6, allows to take under consideration the values, which are laying in close proximity of a granule, but are not a part of it.

Figure 6 b shows changes within the age attribute. The created granules for α = 0 overlap significantly between classes *n* = 1,…,3, thus they provide a little useful information for classification purposes. Moreover, the median calculated for 2nd and 3rd class (*n*) is placed almost in the same place - around 55 years. Nevertheless, the shapes of fuzzy sets for *α* ∈ (0, 1) differ significantly and therefore can be used as information to find the correct fibrosis class. The fuzzy set for *α*_*j*_ =0.5 and *α*_*j*_ =1.0 (Fig. 6 b) provides more information as offering smaller overlap of the sets. To improve classification accuracy the attributes, which sets overlap significantly [33], are removed from further classification. Only selected set of attributes *K'* ⊂ K with the smallest overlap of fuzzy intervals between all fibrosis classes, are processed further.

### Classification process

**Fig. 7:**
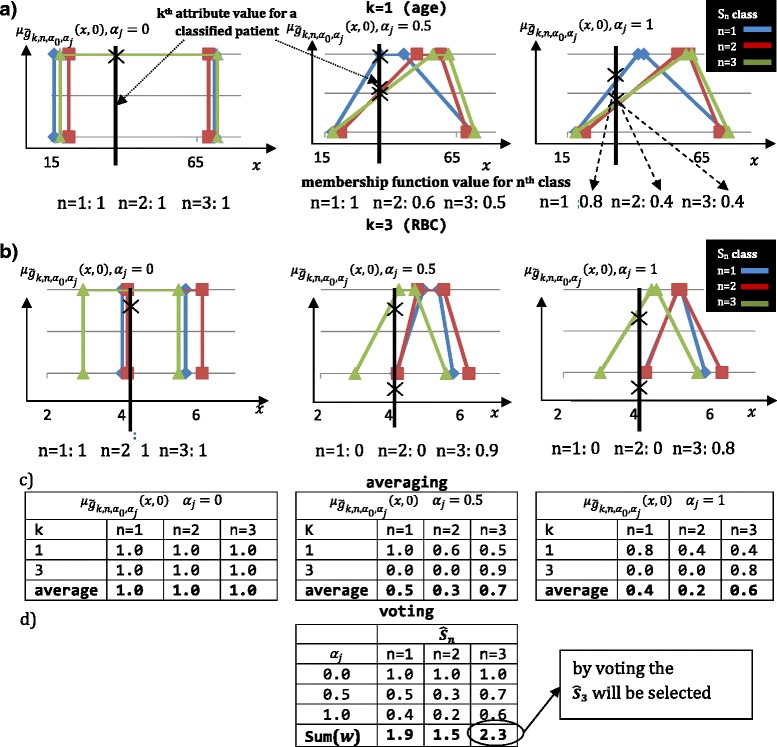
Classification example illustrated on two attributes: Age (**a**) and RBC (**b**). The values of attributes of classified patient are compared against the fuzzy representation of classes. Then, using averaging (**c**) and weighted voting **d**, the patient’s fibrosis class is found

The created granular model is used to evaluate the classes of liver fibrosis for test patients. The data of a given patient is compared with the model using membership function ${\mu}_{{\tilde{g}}_{k,n},0,{\alpha}_j}\left({y}_{k,d}\right)$, where *y*_*k*_ is the medical examination result of a patient for a given *k*^*th*^ attribute. The membership function provides information, whether or not a patient has *n*^*th*^ liver fibrosis class, according to *k*^*th*^ attribute. The aggregation (averaging) over all attributes *(k)* is performed for each *α*_*j*,_*j* = 0.. *z* − 1 and *n*^*th*^ class separately. As a result, the average value for each class is evaluated. Finally, the voting is performed based on the values obtained for each of *z* α-cuts. The motivation of voting was based on the physician analysis scheme, where similarities of symptoms are analyzed. In this case, the classification procedure weight, how many fuzzy granules favor a given class. The aggregation and classification process is illustrated in Fig. 7 for two attributes: age (k = 1) and RBC (k = 3).

The black line in Fig. 7 illustrates the values of attributes of classified patient. If the patient does not have an attribute marked the attribute is not taken into consideration. Using only results for *α*_*j*_ *=* 0 the classification is inconclusive (equal value of membership function for all three classes). For the second and third column (*α*_*j*_ =0.5 and *α*_*j*_ =1) age attribute favors n = 1 class (marked by blue curve), while RBC attribute favors (b / RBC) n = 3 class (marked by green curve). To find the correct diagnosis the mean value of membership function is calculated for given *α*_*j*_ (Fig. 7c). As presented in example average values for *α*_*j*_ *=* 0 carry no information, however the average values for *α*_*j*_ equal 0.5 and 1 favors n = 3 class. Finally, the weighted voting is performed between the results acquired for *α*_*j*_ ∈ {0, 0.5, 1}. The class *n* = 3 with the highest value (*w*) is selected.

The patient’s classification is performed formally based on his medical data set *Y* = {*y*_*k*_, *k* ∈ *K*}, where *y*_*k*_ defines a value of *k*^*th*^ attribute. The weight of *n*^*th*^ fibrosis can be calculated using a following equation:

5 $$ {w}_{z,d,K\hbox{'}}\left(Y,n\right)={\displaystyle {\sum}_{j=0}^{z-1}\frac{{\displaystyle {\sum}_{k\in K\hbox{'}}{\mu}_{{\tilde{g}}_{k,n,0,\alpha j}}\left({y}_k,d\right)}}{card\left(K\hbox{'}\right)},{y}_k\in Y} $$

where: *n* is a number of fibrosis class, *K* ' is set of selected attributes, *card* is cardinality of a set, *z* and *d* are the method parameters.

Finally, the class for which *w* function returns maximal value is treated as the patient’s fibrosis class (*Ŝ*):

6 $$ {\widehat{S}}_{z,d,K\hbox{'}}(Y){=}_{n\in \left\{1,\dots, 3\right\}}^{\mathrm{argmax}\left\{{W}_{z,d,K\hbox{'}}\left(Y,n\right)\right\}} $$

where:

*Y*- a set of laboratory blood test results of the patient,

*n* - represent the liver fibrosis class, defined as n = 1, …, 3,

*z*, *d*, *K'* - parameters of proposed method.

The quality of recognition depends on differences between *w*_*z*,*d*,*K* '_ function values. Significant difference between calculated weights, for various classes, confirms that quality of diagnosis is high.

## Results

After medical consultations 3 classes were selected for representation of 5 liver fibrosis levels - see Table 2.

**Table 2:** Liver fibrosis medical data distribution

Fibrosis class	METAVIR scoring scale	Number of patients (%)
S_1_	F0-F1	129 (44,5 %)
S_2_	F2-F3	102 (35,2 %)
S_3_	F4	59 (20,3 %)
Total		290 (100 %)

**Fig. 8:**
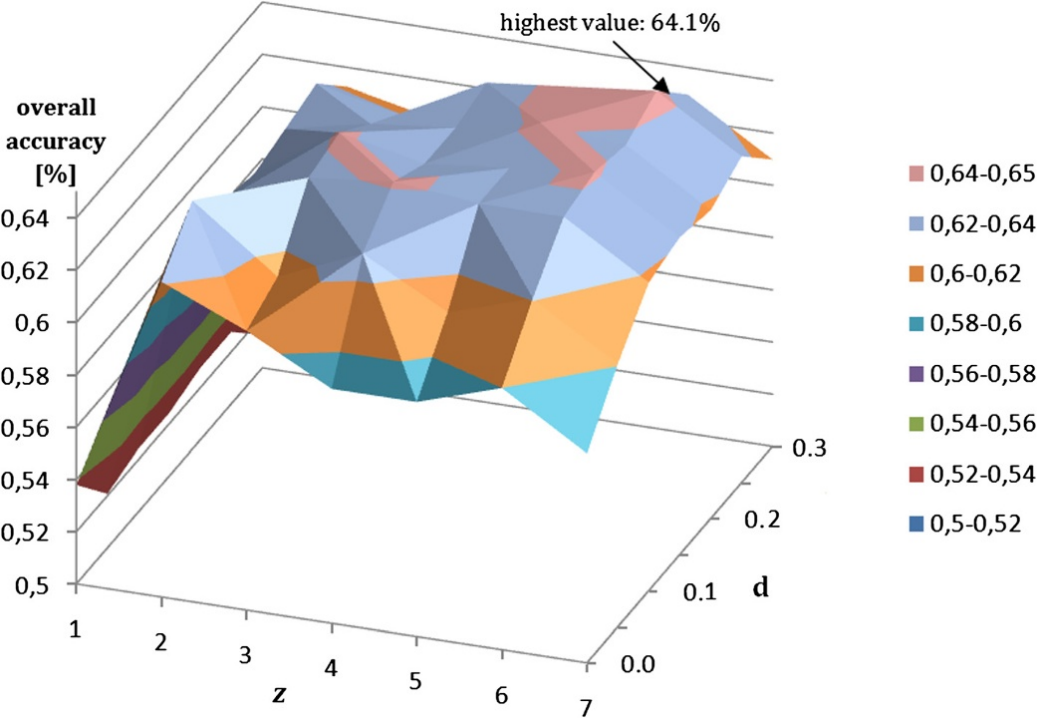
Calibration of proposed method using number of cuts (*z* - discrete parameter) and generalization parameter (*g*)

The number of cuts (*z*) and generalization parameter (*d*) was calibrated using training data and unified sampling. In first case all 26 available attributes were used. The calibration scheme is presented in Fig. 8. It is worth to note that *z* is a discrete parameter.

**Fig. 9:**
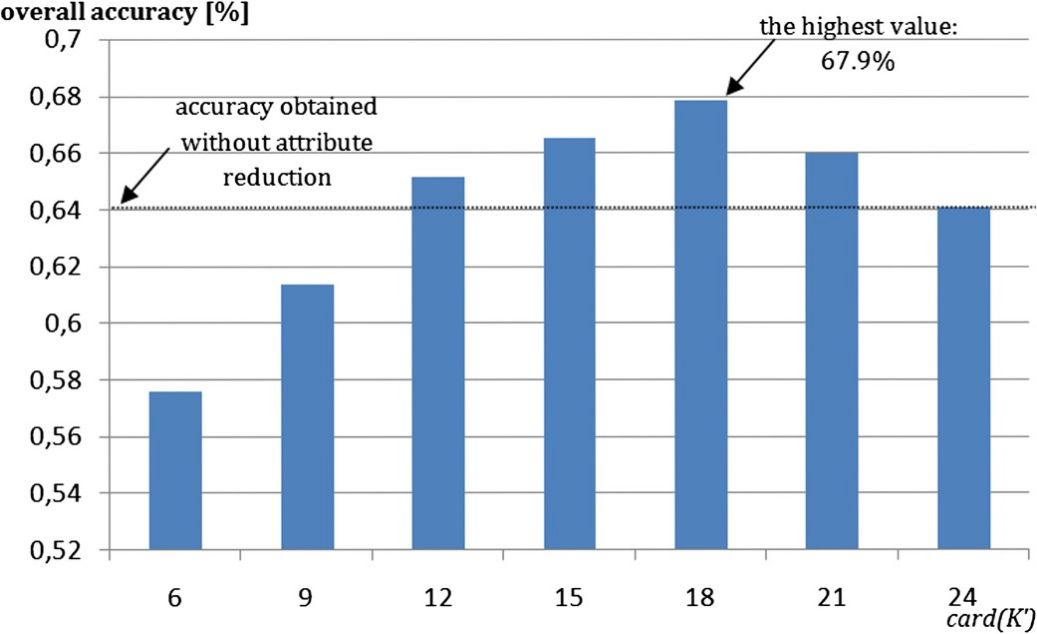
Tuning of proposed method of attributes reduction vs. overall accuracy. The classification is performed only on selected set of attributes (*K'* ⊂K) with the smallest overlap between all fibrosis classes

To improve classifier accuracy, the set of analyzed attributes was decreased to the *K'* set. The dependency between the overall accuracy and number of attributes was analyzed. The results were presented in Fig. 9.

The best result was obtained for card(*K')* = 18. Moreover, for the values from 12 to 21, overall accuracy was improved as well. The attributes reduction, by taking under consideration fuzzy function overlap, improved the overall accuracy by 4 %.

Calibrated model was verified using 10-fold cross validation [30] and the obtained results have been compared with a various, selected classification methods. Results for 290 patients infected with hepatitis C are presented in Tables 3 and 4.

**Table 3:** Results of the proposed method compared to generic classifiers [21, 22, 24, 25]

Method	Overall accuracy [%]	Specificity [%]			Sensitivity [%]		
		*Ŝ*_1_	*Ŝ*_2_	*Ŝ*_3_	*Ŝ*_1_	*Ŝ*_2_	*Ŝ*_3_
Granular model	67.9	62.1	93.1	91.8	86.8	40.2	74.6
Random Tree	61.4	71.4	76.6	90.5	66.7	57.8	55.9
PNN	60.7	70.8	88.8	80.1	76.7	32.4	74.6
Naïve Bayes	59.7	75.8	63.3	96.1	62.0	62.7	49.2
Auto-Binned IBk	58.6	54.0	79.3	97.0	77.5	38.2	52.5
RecBF-DDA	56.9	60.4	72.8	96.2	65.2	53.1	46.6
IBk (k-NN)	49.3	30.4	82.4	99.1	87.6	20.6	15.3

**Table. 4:** Confusion table for Granular Model

	Classified as		
Biopsy	*Ŝ*_1_	*Ŝ*_2_	*Ŝ*_3_
*F0+F1*	112	9	8
*F2+F3*	50	41	11
*F4*	11	4	44

Sensitivity and specificity are statistical measures of the performance of a binary classification test. Specificity (sometimes called the true negative rate) measures the percentage of people who are correctly identified as not having this stage of liver fibrosis. Sensitivity (also called the true positive rate) measures the percentage of people who are correctly identified as having the defined stage of liver fibrosis. Comparing to the proposed method the overall accuracy is highest. But, what is even more important from medical point of view that only 6.5 % of the misclassification occurred between the first and the last class. The research was extended to verify the model against patients with various liver disease etiologies. Therefore, the 365 patients’ dataset was used, where the patients with HBV, HCV as well as nonalcoholic/alcoholic hepatitis were included. The result was presented in Tables 5 and 6. The proposed model proved to be superior over various other classifiers. The results are stable and 67.4 % of overall accuracy was achieved. Furthermore, misclassification between the first and the last class decreased to 6.2 %.

**Table 5:** Results of the proposed method compared to generic classifiers on 365 patients with various etiologies [21, 22, 24, 25]

Method	Overall accuracy [%]	Specificity [%]			Sensitivity [%]		
		*Ŝ*_1_	*Ŝ*_2_	*Ŝ*_3_	*Ŝ*_1_	*Ŝ*_2_	*Ŝ*_3_
Granular model	67.4	84.2	91.3	91.4	84.2	38.5	78.3
Random Tree	63.4	74.8	76.5	92.1	69.1	54.0	67.0
PNN	61.6	75.7	90.9	75.7	67.1	39.3	81.4
Naïve Bayes	60.8	60.2	84.7	92.9	80.1	38.5	59.7
Auto-Binned IBk	58.4	59.8	79.4	94.7	73.9	40.1	57.7
RecBF-DDA	61.4	77.6	83.1	92.5	63.6	48.3	74.2
IBk (k-NN)	44.8	31.5	76.5	100.0	81.5	31.9	0.0

**Table 6:** Confusion table for Granular Model for 365 patients with various etiologies

	Classified as		
Biopsy	*Ŝ*_1_	*Ŝ*_2_	*Ŝ*_3_
*F0+F1*	123	15	8
*F2+F3*	60	47	15
*F4*	15	6	76

In case, when precise fibrosis class (by METAVIR scale) is required, the method can be applied directly without prior grouping to three classes. In research the dataset of 365 patients with various etiologies was used. The result of five class classification was presented in Tables 7 and 8. The overall accuracy of the method is 52.1 %. The Table 8 shows that using five class classifications the uncertainty of the result must be taken under consideration. However, the majority of misclassification cases are usually made within neighboring classes (111 of all 175). Based on METAVIR scale classification a physician can make more precise decision about future treatment. In future, the analysis will be extended to measure the robustness of method against independent cohort of liver patient from another institution.

**Table 7:** The method accuracy considering 5 classes of fibrosis by METAVIR scale

Metavir class (F)	Specificity (%)	Sensitivity (%)
0	88.6	14.2
1	79.5	56.8
2	88.9	22.8
3	93.0	36.9
4	88.0	81.4
Overall accuracy (%):		52.1

**Table 8:** The confusion matrix presented for 5 class of liver fibrosis classification (METAVIR scale) using the proposed method

	Classified as				
Biopsy	0	1	2	3	4
0	3	9	5	2	2
1	25	71	12	6	11
2	7	23	13	10	4
3	2	10	14	24	15
4	5	7	3	3	79

## Conclusions

Obtained results, on a given data set, are promising and proved to be superior to other classifiers. Moreover, the data representation in form of information granules (intervals and fuzzy sets) can be presented graphically, while the conclusion is made by intuitive voting procedure. Unfortunately, direct comparison against commercial methods is currently impossible, due to unavailable borderline comparison database. Neighboring liver fibrosis stages are often hard to distinguish even by liver biopsy, therefore in the presented research, only three classes of liver fibrosis severity have been defined. Nevertheless, if needed, the model can be used to perform full, five classes, METAVIR classification. The overall accuracy measure (number of samples classified correctly to the number of all samples in the set) which has been chosen to rank different methods is a common, but imperfect measure, as preferably all classes in the test set should have equal cardinalities, which is not fulfilled in the presented case. The proposed method tackled well with uneven classes and missing data, performing balanced diagnosis with relatively high accuracy. The strong points of the method are: use of routine blood tests, good performance on a small subset of parameters, easy principles, and repetitiveness of results (not using random factors).

## Abbreviations

HCV: Hepatitis C virus infection
WHO: World Health Organisation
METAVIR: Inflammation and fibrosis evaluation system used to assess of a liver biopsy of patients with hepatitis C blood attributes
RBC: Red blood cells
WBC: White blood cells
PTL: Platelets
PT: Prothrombin time
PTP: Prothrombin ratio
APTT: Activated partial thromboplastin time
INR: International normalised ratio
AST: Aspartate aminotransferase
ALT: Alanine aminotransferase
ALP: Alkaline phosphatse
GGT: γ-glutamyltransferase.
Na: Natrium
K: Kalium
PNN: Probabilistic Neural Network
IBk: k-nearest neighbours classifier implementation without distance weighting
RecBF-DDA: Radial Basis Function network with Dynamic Decay Adjustment
